# Classification and identification of agricultural products based on improved MobileNetV2

**DOI:** 10.1038/s41598-024-53349-w

**Published:** 2024-02-11

**Authors:** Haiwei Chen, Guohui Zhou, Wei He, Xiping Duan, Huixin Jiang

**Affiliations:** 1 School of Computer Science and Information Engineering, Harbin Normal University, Harbin, 150025 China; 2 School of Life Sciences and Technology, Harbin Normal University, Harbin, 150025 China

**Keywords:** Computer science, Agroecology

## Abstract

With the advancement of technology, the demand for increased production efficiency has gradually risen, leading to the emergence of new trends in agricultural automation and intelligence. Precision classification models play a crucial role in helping farmers accurately identify, classify, and process various agricultural products, thereby enhancing production efficiency and maximizing the economic value of agricultural products. The current MobileNetV2 network model is capable of performing the aforementioned tasks. However, it tends to exhibit recognition biases when identifying different subcategories within agricultural product varieties. To address this challenge, this paper introduces an improved MobileNetV2 convolutional neural network model. Firstly, inspired by the Inception module in GoogLeNet, we combine the improved Inception module with the original residual module, innovatively proposing a new Res-Inception module. Additionally, to further enhance the model's accuracy in detection tasks, we introduce an efficient multi-scale cross-space learning module (EMA) and embed it into the backbone structure of the network. Experimental results on the Fruit-360 dataset demonstrate that the improved MobileNetV2 outperforms the original MobileNetV2 in agricultural product classification tasks, with an accuracy increase of 1.86%.

## Introduction

Detecting and classifying agricultural products are fundamental means to maximize the economic value of agricultural produce. With the maturity and development of technologies such as computer image processing and deep learning, the process of agricultural product sorting, which is a crucial step in the deep processing of agricultural products, is gradually being replaced by automated machines^1^. By employing machine vision detection and automatic classification of agricultural products, we can not only avoid the issues associated with low efficiency, product damage, and varying classification standards in manual sorting but also enhance classification accuracy. This, in turn, contributes to the sustainable development of agriculture^2^.

The accuracy of agricultural product identification is crucial for the agricultural and food industries^3^. On one hand, the accuracy of agricultural product identification directly impacts quality control. Inaccurate identification may lead to erroneous quality assessments, thereby affecting the market competitiveness of agricultural products^4^. On the other hand, in the food supply chain, accurate identification of agricultural products is a key factor in achieving traceability. If identification is not accurate, it may result in difficulties in product traceability, making the trace-back and recall of problematic products more complex. Lastly, the accuracy of agricultural product identification is also vital for business analysis and decision-making. Accurate identification provides agricultural enterprises with more precise data, aiding in the formulation of scientifically informed business strategies^5^.

In recent years, many experts have introduced various algorithms for the automatic classification of agricultural products^6^. For example, Kang et al.^7^ developed and employed a lightweight backbone network called LedNet, combined with a feature pyramid network and an untracked spatial pyramid pool to enhance the model’s detection performance. In apple detection in orchards, they achieved a recall rate of 82.1% and an accuracy of 85.3%. Chen et al.^8^ proposed a fruit image classification method based on multiple optimized convolutional neural networks. They first used wavelet threshold denoising on fruit images, followed by gamma transformation for image correction, and then introduced a SOM network for sample pre-learning, achieving accuracy of up to 99%.Costa et al.^9^ constructed a dataset containing 43,843 tomatoes with external defects and used fine-tuned ResNet50 for tomato defect detection, achieving an average accuracy of 94.6% on the test set. MS et al.^10^ proposed two different deep learning frameworks, with the better-performing one being a fine-tuned Visual Geometry Group-16 pretrained deep learning model, which achieved an accuracy of 96.75% on challenging fruit images. Rehman et al.^11^ used transfer learning to train the model, then enriched the feature set through feature fusion, and finally optimized it using the Whale Optimization Algorithm (WOA) to classify six different diseases of citrus plants, achieving an accuracy of 95.7%. Nasiri et al.^12^ proposed a method to distinguish healthy dates from defective ones, using a CNN model built with VGG-16. Through model optimization, they achieved an accuracy of 96.98%.

In comparison, computer vision and multi-class support vector machines (SVM) have been used to classify different varieties of fruits, achieving an accuracy of 88.20%^13^. Siddiqi et al.^14^ studied fruit image classification based on the Inception v3 and VGG16 models, including transfer learning and fine-tuning. Experimental results showed that they could achieve an accuracy of 99.27% across 72 categories in the Fruit 360 dataset. Ghosh et al.^15^ utilized the same pretrained convolutional neural network, ShuffleNetV2, and constructed convolutions with more feature channels. Their model reached an accuracy of 96.24% on 41 categories in the Fruit 360 dataset.

These research results demonstrate that methods for the automatic classification of agricultural products have made significant advancements in recognizing different types of fruits and have played a crucial role in improving classification accuracy. Previous studies have shown that models capable of extracting features more finely perform better in multi-class tasks, especially as the number of fruit categories to classify increases. In this regard, the Inception architecture of the GoogLeNet model^16^ has been instrumental in extracting features from feature maps using convolutional kernels of different sizes, and networks equipped with such modules often exhibit superior performance. For instance, Yang et al.^17^ improved the GoogLeNet model, with a primary focus on the Inception module. The enhanced model achieved a recognition accuracy of 99.58% in identifying diseases in rice leaves. On the other hand, Husaini et al.^18^ constructed Inception V3, Inception V4, and an improved version called Inception MV4. These models significantly improved the recognition performance for breast cancer. In summary, the Inception modules, as seen in models like GoogLeNet, have been pivotal in enhancing feature extraction capabilities and improving classification accuracy across various domains, including agriculture.

With the success of Transformers in natural language processing^19^, attention mechanisms have also been introduced to the computer vision domain. For instance, the recently introduced Efficient Multi-scale Attention (EMA) module^20^ supports cross-spatial learning and has significantly improved performance in tasks such as image classification and object detection.

It should be noted that most research papers often focus on the classification of specific fruits and less on the classification of different fruits. For example, in the Fruit-360 dataset^21^, which contains 131 different fruit categories, there are few studies attempting to classify all these fruits simultaneously. This is primarily due to the diversity of fruit types, and testing each type of fruit would significantly increase the time and cost of research. Considering that agricultural product recognition and classification will inevitably be performed on embedded devices, MobileNetv2, as a lightweight convolutional neural network architecture, is well-suited for such scenarios.

Therefore, this study introduces an innovative approach by applying an improved version of the MobileNetv2^22^ model to the Fruit-360 dataset, aiming to comprehensively train and classify all 131 fruit classes at once. This initiative is designed to address the shortcomings in existing research and provide a more comprehensive solution for classifying different fruits. The main contributions of this study can be summarized as follows:

1. The introduction of the Res-Inception module, which combines residual and Inception modules, has been implemented to better extract features and achieve improved classification results for all 131 categories in the Fruit-360 dataset.
2. Inspired by the Transformer concept, we introduced the Efficient Multi-scale Attention (EMA) module for cross-spatial learning, which has a significant impact on improving recognition accuracy.
3. We compared our model's accuracy with state-of-the-art algorithms, considering its relatively fewer parameters, making it suitable for most embedded devices, and achieved an impressive accuracy of up to 99.96%.

## Related work

### Multiclass recognition

In the preceding literature, the primary focus has been on the recognition of a limited number of agricultural product categories. In Ref.^23^, an integrated model was introduced, combining bottleneck features from two multitask deep convolutional neural networks (ResNet-50 and ResNet-101). However, this multitask framework included only two branches dedicated to fruit recognition. In Ref.^24^, a fruit recognition algorithm based on convolutional neural networks (CNN) was proposed. Initially, the Selective Search algorithm was employed to extract image regions, followed by the use of entropy from fruit images to select specific areas. These regions were then utilized as inputs for training and recognition within the CNN neural network. Despite achieving certain success in fruit recognition, this method still exhibits limitations in recognizing a diverse range of fruit categories. In Ref.^25^, a novel artificial intelligence system was presented for fruit classification. The approach involved the extraction of features from fruit images using two-dimensional fractional Fourier entropy with a rotation angle vector grid. Subsequently, a five-layer stacked sparse autoencoder was employed as the classifier. The system demonstrated significant success on an 18-class fruit dataset, achieving a noteworthy 95.08% micro-average F1 score. Considering CNN and transfer learning approaches, this study^26^ proposed an effective date classification model and created a dataset containing eight different categories of date fruits for model training. In Ref.^27^, an automatic pineapple classification method was introduced. This method utilized an embedded onboard computing processor, servos, and ultrasonic sensors to create a knocking machine integrated with a conveyor belt for automatic separation of pineapples. Concurrently, the performance of a convolutional neural network (CNN) based on deep learning was tested, with the developed CNN model achieving an optimal accuracy rate of 0.97. These studies underscore the challenges in achieving comprehensive recognition across a wide variety of agricultural product categories. Therefore, this paper opts for the Fruit-360 dataset, encompassing 131 fruit classes, to address the need for a model capable of recognizing a diverse range of fruits in various scenarios.

### Recognition of agricultural product subcategories

**Figure 1:**
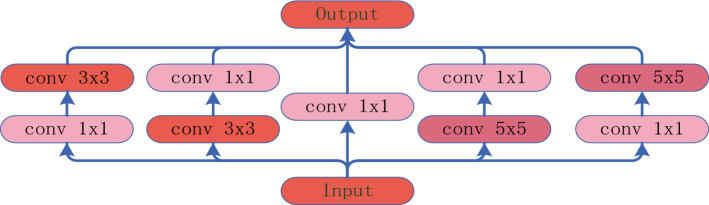
Inception specific structure.

Accurate recognition of subcategories within agricultural products is widely recognized as a challenging task in the field of image recognition. Due to the diversity of agricultural products, a single type may encompass multiple varieties or subtypes, making fine-grained classification a complex and crucial undertaking. One primary challenge of existing models^28^ in agricultural product classification is their poor performance in handling subtypes with similar features. To address this issue, we introduce an efficient multi-scale cross-space learning module with attention (EMA) and an Inception module to enhance the accuracy of recognizing subcategories within agricultural products. The efficient multi-scale cross-space learning module (EMA) reorganizes certain channels into batch processing dimensions and divides channel dimensions into multiple sub-features to ensure the even capture of spatial semantic features within each feature group. The EMA mechanism utilizes excitation to assess the importance of different parts of the input data for the current task and uses modulation to adjust the weights of these parts, optimizing the model's performance. The advantage of this mechanism lies in its ability to extract important information relevant to the current task, thereby reducing interference from irrelevant information and improving fine-grained classification capabilities. The Inception module, as shown in Fig. 1, captures different image features by simultaneously using multiple scales of convolutional kernels and max-pooling layers. This enables the network to learn across multiple feature scales.

### Real-time processing and efficiency

**Figure 2:**
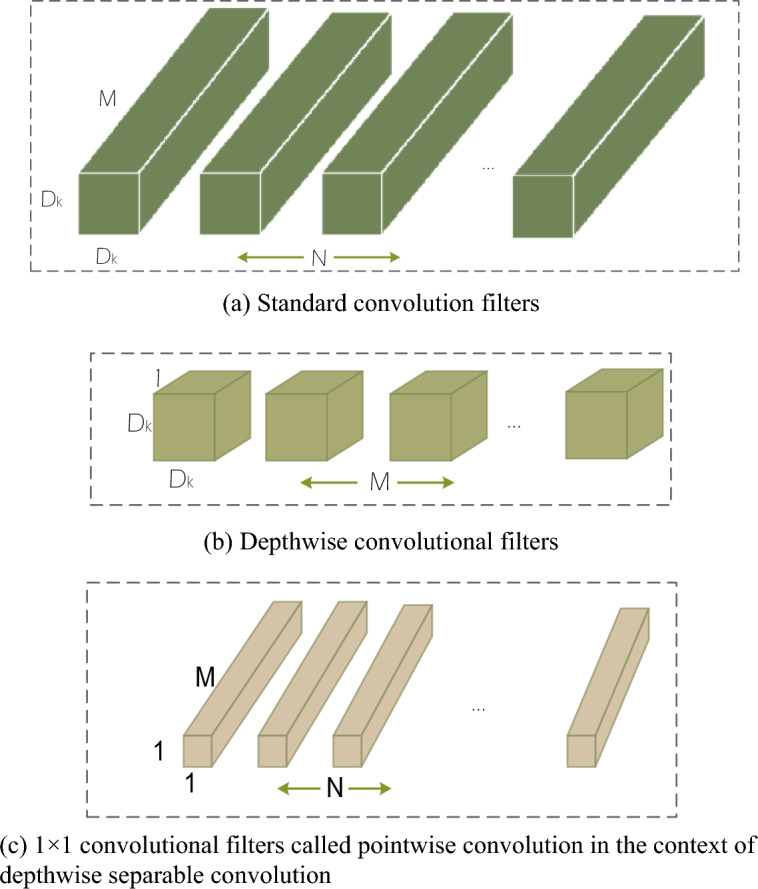
The conventional convolutional filters shown in (**a**) have been substituted with two distinct layers: depthwise convolution as illustrated in (**b**) and pointwise convolution as depicted in (**c**). ${\text{D}}_{{\text{k}}}$ is the spatial dimension of the kernel assumed to be square and ${\text{M}}$ is the number of input channels and ${\text{N}}$ is the number of output channels.

In the context of agricultural product harvesting, real-time processing and efficiency are critical concerns^29^. In scenarios with limited resources, some models may require more computational resources, thereby limiting their feasibility in practical applications. Particularly, larger models may not perform well in such resource-constrained situations. To address this issue, the development of lightweight models has made significant progress in recent years, with the MobileNet series being a prominent highlight^30^. MobileNetv2, as a lightweight convolutional neural network architecture, is specifically designed to operate in resource-constrained environments such as mobile devices and embedded systems. It has been widely applied in image recognition and computer vision tasks. One key feature of this network is depthwise separable convolution, as illustrated in Fig. 2. It decomposes standard convolution operations into two steps: depthwise convolution and pointwise convolution. This reduces computational costs while effectively capturing image features. Additionally, MobileNetv2 introduces an inverted residual structure, enhancing the network's non-linearity and making it more suitable for various image features, especially in edge cases and low-quality images. The design focus of MobileNetv2 is on lightweight characteristics, making it an ideal choice for embedded devices and mobile applications. In the agricultural domain, particularly in the context of agricultural product harvesting, the application of such lightweight models contributes to improving real-time processing and efficiency while overcoming limitations posed by limited computational resources.

## Methodologies

### Overall framework

**Figure 3:**
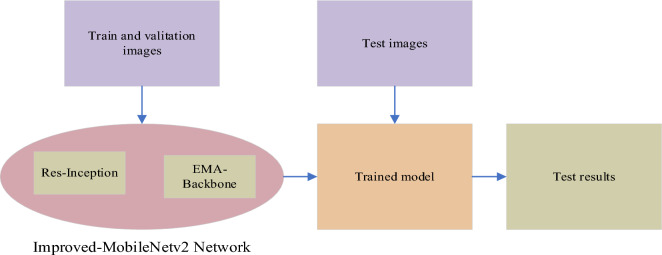
The overall framework of the model.

As shown in Fig. 3, we divided the dataset into a training set, a validation set, and a test set. During the model training phase, we utilized the training set and the validation set to train the Improved-MobileNetv2 model, resulting in a trained model. During the testing phase, we directly tested the test set using this model.

### Improved-MobileNetv2

**Figure 4:**
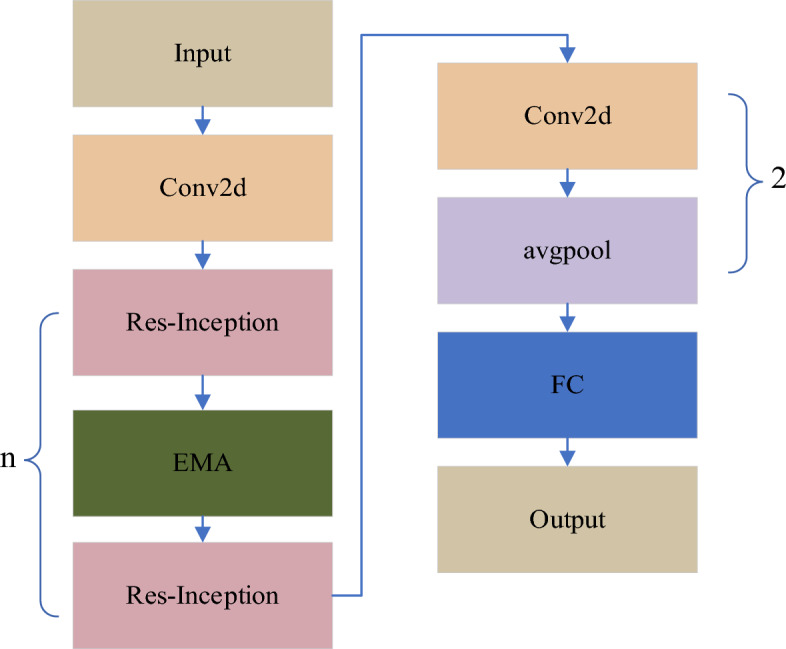
Improved MobileNetv2 framework.

When dealing with an increased number of agricultural product categories, especially in classifying subcategories, existing models, particularly the original MobileNetv2, have some limitations in accuracy. This can lead to errors in recognizing different types of agricultural products by automated equipment, reducing production efficiency, and causing economic losses. To address this issue, we propose an agricultural product recognition model based on the improved MobileNetv2. As shown in Fig. 4, the improved model primarily focuses on the backbone architecture, which is the core part of the model. By enhancing the network's feature extraction capabilities, we aim to improve the accuracy of subcategory recognition. We introduce the improved Res-Inception module to replace the original residual module. Additionally, between every two modules, we introduce an efficient multi-scale spatial attention module, implementing cross-space learning (EMA). This improvement aims to enhance the model's accurate recognition capability of agricultural product subcategories. Firstly, comparative experiments on the Fruit-360 dataset demonstrate that our improved model achieves higher recognition accuracy. Subsequently, through ablation experiments on different modules of the improvement, we can observe the impact of our improved modules on the model's accuracy. Through these two experiments, we conclude that our improved MobileNetv2 network achieves superior performance and detection accuracy. This optimization is expected to better adapt to the recognition tasks of different agricultural product subcategories in complex scenarios. Below is a detailed introduction to the two improved modules.

#### Res-Inception module

**Figure 5:**
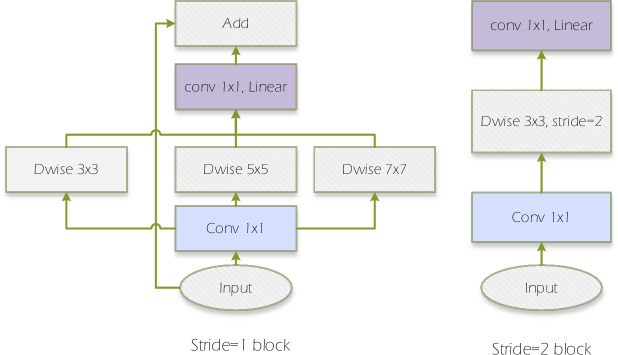
Res-Inception module.

Inspired by the Inception structure, we introduced two larger convolutional kernels, namely Dwise 5 × 5 and Dwise 7 × 7, into the depthwise separable convolution model, as shown on the left side of Fig. 5. This improvement aims to enhance our model's capability to extract feature information. When the stride (Stride) is set to 1, the model will execute the module on the left. To avoid the issue of the model becoming overly deep, which could potentially decrease its performance, we introduced a connection between the input and output of this module. When the stride is not equal to 1, the model will continue to execute the original depthwise separable convolution module. This design allows us to extract more feature information while effectively controlling the convolutional depth, thereby improving the model's performance.

#### Backbone network with EMA

**Figure 6:**
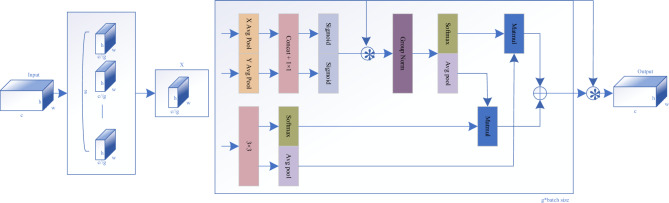
EMA structure diagram. Where g represents the number of groups, X Avg Pool represents one-dimensional horizontal pooling operation, and Y Avg Pool represents one-dimensional vertical pooling operation.

We embed the EMA module into the backbone network of the model. EMA primarily involves partitioning channels into several sub-groups and assigning different weights to different regions of the feature maps within these sub-feature groups, as shown in Fig. 6. This module first divides the input feature map $x \in {\mathbb{R}}^{c \times h \times w}$ into g groups along the channel dimension, with g set to 8 in this experiment, to learn different semantics, denoted as groups and represented as $x = [x_{0} ,x_{1} ,...,x_{g - 1} ],x_{i} \in {\mathbb{R}}^{{c//g \times {\text{h}} \times w}}$.

As shown in Fig. 6, EMA employs three parallel paths to extract features from groups. Two of these paths use 1 × 1 convolutions, while the third path employs a 3 × 3 convolution. In the two 1 × 1 convolution paths, we perform one-dimensional horizontal pooling and one-dimensional vertical pooling, followed by aggregating the outputs of both channels through simple multiplication. This results in two tensors, one from the 1 × 1 branch and the other from the 3 × 3 branch. Next, we utilize 2D global average pooling to encode global spatial information from the output of the 1 × 1 branch, while transforming the output of the 3 × 3 branch into the corresponding dimension shape, denoted as ${\mathbb{R}}_{1}^{1 \times c//g} \times {\mathbb{R}}_{3}^{c//g \times hw}$^31^. The formula for the 2D global pooling operation is represented as follows.

1 $$z_{c} = \frac{1}{h \times w}\sum\limits_{j}^{h} {\sum\limits_{i}^{w} {x_{c} \left( {i,j} \right)} } .$$

By performing a matrix dot product operation on the outputs from the parallel processing described above, we obtain the first spatial attention map. Similarly, we employ 2D global average pooling to encode global spatial information from the 3 × 3 branch, while the 1 × 1 branch is directly transformed into the corresponding dimension shape before the joint activation mechanism of channel features, denoted as ${\mathbb{R}}_{3}^{1 \times c//g} \times {\mathbb{R}}_{1}^{c//g \times hw}$. Then, we obtain the second spatial attention map, preserving complete and precise spatial positional information. Subsequently, the output feature maps within each group are computed as an aggregation of the two generated spatial attention weight values, followed by passing through a Sigmoid function. This process captures pixel-level pairwise relationships and emphasizes global context for all pixels. The final output of EMA is of the same size as the input x, which is highly efficient and effective for stacking into modern architectures.

## Experiments

### Experimental details

#### Datasets

Fruit-360 is a dataset containing 90,483 fruit photographs. As shown in Table 1, the series contains 131 different types of fruits of different species^32^. The size of these pictures is $100 \times 100$ pixels. Because of the needs of the model, further processing of this dataset will be described in the subsection on graphical processing. These images were obtained by shooting a short twenty-second video of the fruit while it was slowly rotated by a motor, and then extracting frames/images from that movie. White paper is being used as a background for capturing an image of fruits. The algorithm is then applied to eliminate the background of each fruit. This is important to ensure that the data can be easily accessed and used by other researchers, promoting transparency and reproducibility in scientific research.

**Table 1:** Datasets of fruit-360.

Fruit types	No. of images	Fruit types	No. of images	Fruit types	No. of images
Apple Braeburn	656	Grape blue	1312	Pear Monster	656
Apple Crimson snow	592	Grape pink	656	Pear red	888
Apple golden 1	640	Grape white	656	Pear stone	948
Apple golden 2	656	Grape white 2	656	Pear Williams	656
Apple golden 3	642	Grape white 3	656	Pepino	656
Apple Granny Smith	656	Grape white 4	629	Pepper green	592
Apple pink lady	608	Grapefruit pink	656	Pepper orange	936
Apple red 1	656	Grapefruit white	656	Pepper red	888
Apple red 2	656	Guava	656	Pepper yellow	888
Apple red 3	573	Hazelnut	621	Physalis	656
Apple red delicious	656	Huckleberry	656	Physalis with husk	656
Apple red yellow 1	656	Kaki	656	Pineapple	656
Apple red yellow 2	891	Kiwi	622	Pineapple mini	656
Apricot	656	Kohlrabi	628	Pitahaya red	656
Avocado	570	Kumquats	656	Plum	598
Avocado ripe	657	Lemon	656	Plum 2	562
Banana	656	Lemon Meyer	656	Plum 3	1204
Banana lady finger	602	Limes	656	Pomegranate	656
Banana red	656	Lychee	656	Pomelo sweetie	603
Beetroot	600	Mandarine	656	Potato red	600
Blueberry	616	Mango	656	Potato red washed	604
Cactus fruit	656	Mango Red	568	Potato sweet	600
Cantaloupe 1	656	Mangostan	402	Potato white	600
Cantaloupe 2	656	Maracuja	656	Quince	656
Carambula	656	Melon Piel de Sapo	984	Rambutan	656
Cauliflower	936	Mulberry	656	Raspberry	656
Cherry 1	656	Nectarine	656	Redcurrant	656
Cherry 2	984	Nectarine flat	640	Salak	652
Cherry rainier	984	Nut forest	872	Strawberry	656
Cherry wax black	656	Nut Pecan	712	Strawberry wedge	984
Cherry wax red	656	Onion red	600	Tamarillo	656
Cherry wax yellow	656	Onion Red Peeled	595	Tangelo	656
Chestnut	603	Onion white	584	Tomato 1	984
Clementine	656	Orange	639	Tomato 2	897
Cocos	656	Papaya	656	Tomato 3	984
Corn	600	Passion fruit	656	Tomato 4	639
Corn husk	616	Peach	656	Tomato cherry red	656
Cucumber ripe	522	Peach 2	984	Tomato heart	912
Cucumber ripe 2	624	Peach flat	656	Tomato Maroon	494
Dates	656	Pear	656	Tomato yellow	632
Eggplant	624	Pear 2	928	Tomato not ripened	612
Fig	936	Pear Abate	656	Walnut	984
Ginger root	396	Pear Forelle	936	Watermelon	632
Granadilla	656	Pear Kaiser	402	Total	90,483

#### Evaluation indicators

**Figure 7:**
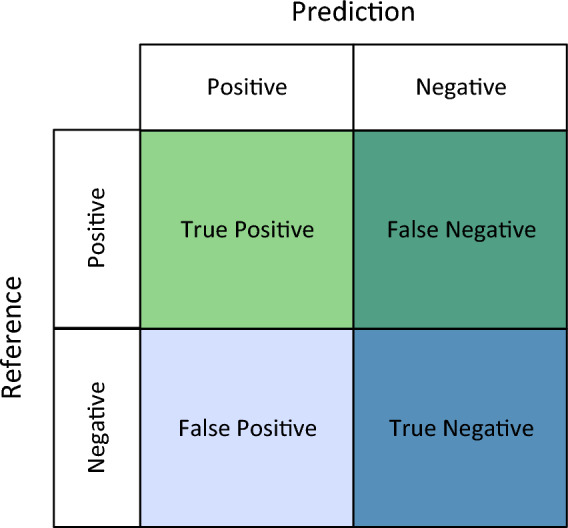
Confusion matrix.

In this study, the performance of the model is presented in the form of a variant of the $2 \times 2$ confusion matrix. as shown in the Fig. 7.

Precision, recall and accuracy are used to evaluate the performance of the network model for fruit recognition. The precision, recall and accuracy rates are calculated as follows:

Precision: A metric that measures the proportion of samples that were predicted as positive by the model and were actually positive, out of all the samples predicted as positive.

2 $$\Pr ecision = \frac{TP}{{TP + FP}}.$$

Accuracy: A metric that measures the proportion of correctly classified samples out of the total number of samples.

3 $$Accuracy = \frac{TP + TN}{{TP + FN + FP + TN}}.$$

Recall: A metric that measures the proportion of samples that were correctly predicted as positive by the model, out of all the samples that were actually positive.

4 $${\text{Re}} call = \frac{TP}{{TP + FN}}.$$

F1 score: A metric that provides a balanced evaluation of a model's performance by combining both precision and recall. It is the harmonic mean of precision and recall, and represents the overall accuracy of the model in identifying positive samples.

5 $$F1 = \frac{2TP}{{2TP + FP + FN}}.$$

In which TP represents the true positives (the number of target frames correctly predicted as belonging to the positive class), FP represents false positives (the number of target frames incorrectly predicted as belonging to the positive class), and FN represents false negatives (the number of target frames that actually belong to the positive class but are incorrectly predicted as belonging to the negative class).

#### Experimental setup

The software environment used in the experiments includes TensorFlow 2.10 and Python 3.9. The computer configuration is as follows: 12th generation Intel(R) Core(TM) i7-12650H 2.30 GHz processor; 48.0 GB of RAM; NVDIA GeForce RTX 4060 laptop GPU; Cuda 11.3.1 and Cudnn 8.2.1. All experiments were conducted for 100 epochs with a batch size of 32. During the model training process, we employed the Adam optimizer and the cross-entropy loss function, with all other parameters set to their default values.

### Experimental design

To quantitatively evaluate the performance of the improved model, we conducted tests on the Fruit-360 dataset. We performed ablation experiments to assess the importance of the Res-Inception and EMA modules within the model, providing deeper insights into their impact on model performance. In order to demonstrate the versatility of our proposed model, we conducted experiments on different datasets. Additionally, we compared our proposed model with state-of-the-art classification frameworks to demonstrate that our proposed classification framework outperforms other popular object detection frameworks in terms of accuracy.

### Experimental results obtained on Fruit360 using an improved-MobileNetv2

**Figure 8:**
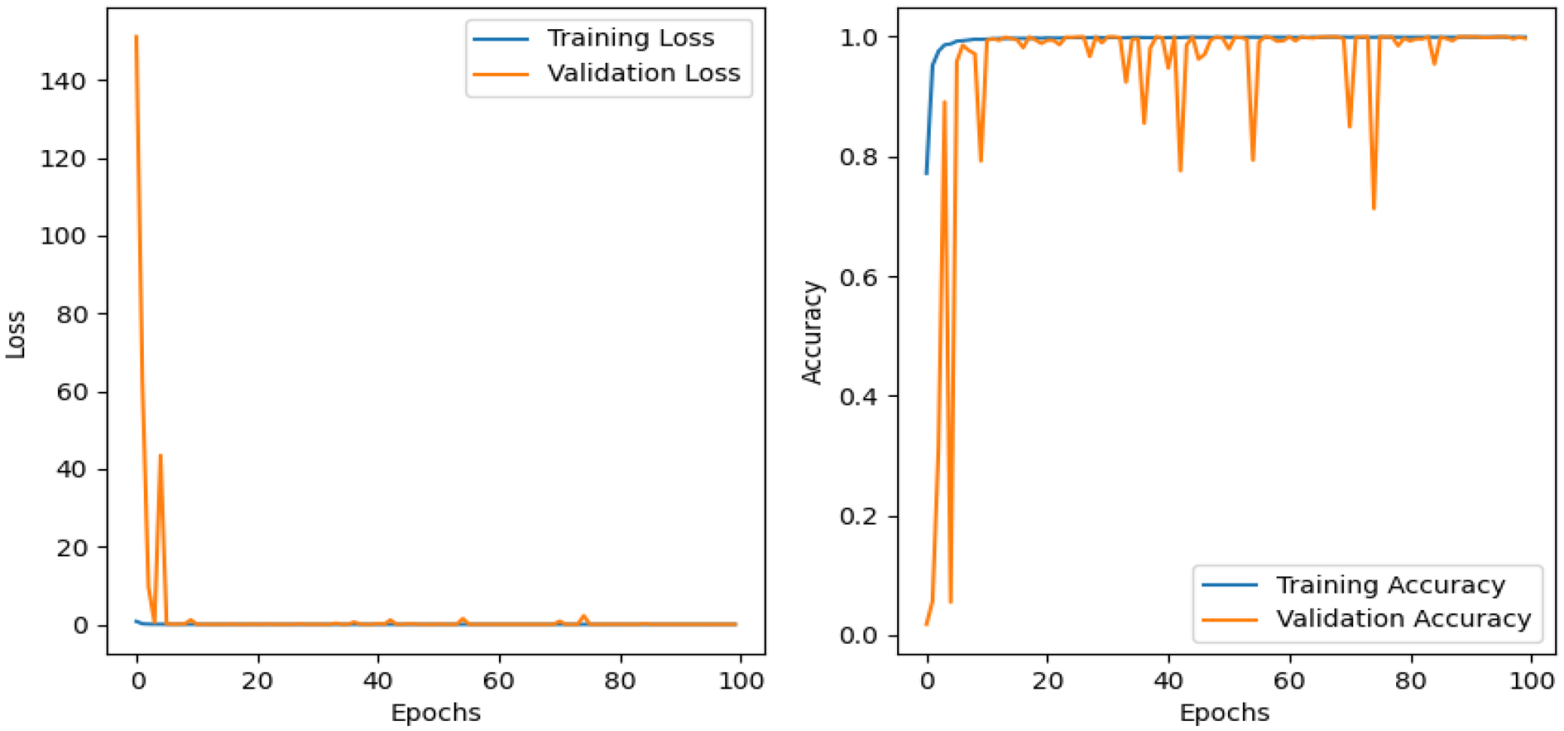
Experimental results of the improved MobileNetv2.

This paper conducted model training on the Fruit360 dataset for 100 epochs and obtained the training results as shown in Fig. 8. The left side of Fig. 8 displays the loss curve of the improved MobileNetv2 model, which reflects the predictive performance of the algorithm regarding target quality. The horizontal axis represents training epochs, and the vertical axis represents the overall loss. Observing the graph, it is evident that the overall loss rapidly decreases during training and stabilizes after around 80 epochs. These results indicate that the improved MobileNetv2 model exhibits excellent convergence performance. The right side of Fig. 8 presents the accuracy on the training set and validation set, with the horizontal axis representing epochs and the vertical axis representing accuracy. It can be observed that the accuracy on the training set steadily increases, while the validation set exhibits some fluctuations in the first 80 epochs but stabilizes afterward. This suggests that our improved model is well-suited for agricultural product classification tasks. Because the heatmap is too large, it is divided into 6 generated in the attached, the first 5 maps have 22 categories each and the last map has 21 categories.

### Ablation experiments

Table 2 presents the results of ablation experiments conducted on the Improved-MobileNetv2 model. The experiments involved four different configurations: the baseline configuration, enabling only the Res-inception module, enabling only the EMA (Excitation and Modulation Attention) module, and enabling both the Res-inception module and the EMA module simultaneously. The results show that enabling either the Res-inception module or the EMA module has a positive impact on the model's performance. Particularly, when both the Res-inception module and EMA module are enabled simultaneously, the model performs the best, achieving the highest F1 score (99.97%), precision (99.96%), and overall accuracy (99.97%). This indicates that the combination of the Res-inception module and EMA module is crucial for improving the accuracy and performance of the Improved-MobileNetv2 model in agricultural product classification tasks.

**Table 2:** Improved-MobileNetv2 ablation experiments.

Res-inception	EMA	F1 scores	Precision	Accuracy
		98.20%	97.43%	98.11%
✓		98.30%	97.69%	99.23%
	✓	98.34%	98.46%	99.47%
✓	✓	99.97%	99.96%	99.97%

**Figure 9:**
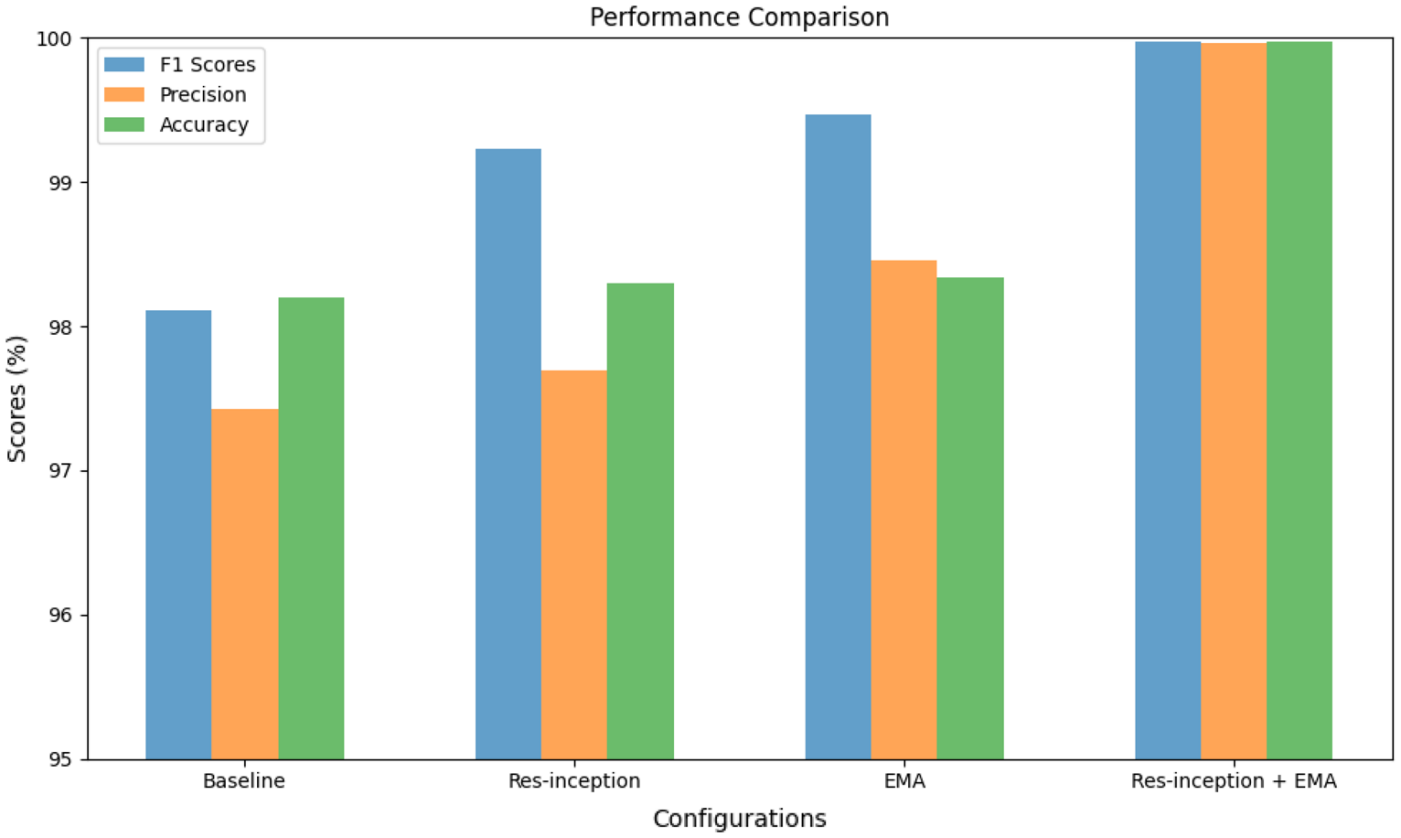
Ablation experiments of improved-MobileNetv2.

As shown in Fig. 9, enabling each module leads to a certain degree of performance improvement in the model. Particularly, when both the Res-inception and EMA modules are enabled simultaneously, the model’s performance reaches its optimal state. Therefore, the concurrent application of these modules proves to be highly beneficial for the enhancement of our model.

### Comparative experiments

Higher accuracy significantly improves production efficiency and increases economic returns in the automated agricultural product classification process. Therefore, this section compares the accuracy of the improved model with the original MobileNetV2 model. Additionally, we compare the accuracy of the improved model with that of AlexNet^33^, VGG16^34^, ResNet34, ResNet50^35^, emphasizing further the advantages of the improved model.

#### Comparison the results of different datasets with experiments

The improved MobileNetv2 model is suitable for agricultural product classification. In order to explore the model's generalization capability, we selected fruit recognition and fruit and vegetable image recognition datasets from the Kaggle Datasets. We conducted training and testing on both the MobileNetv2 model proposed in this study and the enhanced MobileNetv2 model. The fruit recognition dataset consists of 23 different categories of fruits, totaling 44,406 images with resolutions of and. This dataset includes images of various fruits typically found in supermarkets and fruit shops, making it highly relevant for supermarket applications. The fruit and vegetable image recognition dataset comprises 36 types of fruits and vegetables, with a total of 3115 images collected from Bing Image Search. This dataset encompasses a wide variety of fruit and vegetable images, including those harvested in fields, picked from trees, and freshly harvested. Such diversity is advantageous for visual applications, particularly in the context of robotic fruit picking. By training and testing the models on these datasets, we can assess their performance and generalization ability across different agricultural product classification tasks.

Table 3 provides a performance comparison between MobileNetv2 and Improved-MobileNetv2 models on two different datasets. Firstly, for the "Fruit Recognition" dataset, Improved-MobileNetv2 outperforms MobileNetv2 significantly across all performance metrics. It achieves an impressive 99.62% precision, 99.61% recall, and an astonishing 99.59% F1 score, with an overall accuracy of 99.62%. In contrast, MobileNetv2 exhibits slightly lower performance on the same dataset with a precision of 97.32%, recall of 97.24%, an F1 score of 96.49%, and an overall accuracy of 97.26%. This demonstrates the significant performance improvement achieved by Improved-MobileNetv2 in the "Fruit Recognition" task. On the "Fruits and Vegetables Image Recognition" dataset, we again observe the outstanding performance of Improved-MobileNetv2. It attains a precision of 97.01%, recall of 96.58%, an F1 score of 96.54%, and an overall accuracy of 96.46%. In comparison, MobileNetv2 performs less impressively on the same dataset with a precision of 95.32%, recall of 95.01%, an F1 score of 94.69%, and an overall accuracy of 95.03%. This indicates that Improved-MobileNetv2 excels in the "Fruits and Vegetables Image Recognition" task as well. In summary, Improved-MobileNetv2 demonstrates outstanding classification performance on both datasets, surpassing the MobileNetv2 model significantly. Particularly in the "Fruit Recognition" task, the performance improvement achieved by the proposed model enhancements is notably remarkable. This underscores the significant importance of the model improvements presented in this study for enhancing the accuracy and performance of agricultural product classification.

**Table 3:** Comparison of improved-MobileNetv2 and MobileNetv2 experimental results.

Model	Dataset	Precision	Recall	F1	Accuracy
MobileNetv2	Fruit recognition	97.32%	97.24%	96.49%	97.26%
	Fruits and vegetables image recognition	95.32%	95.01%	94.69%	95.03%
Improve-MobileNetv2	Fruit recognition	99.62%	99.61%	99.59%	99.62%
	Fruits and vegetables image recognition	97.01%	96.58%	96.54%	96.46%

**Figure 10:**
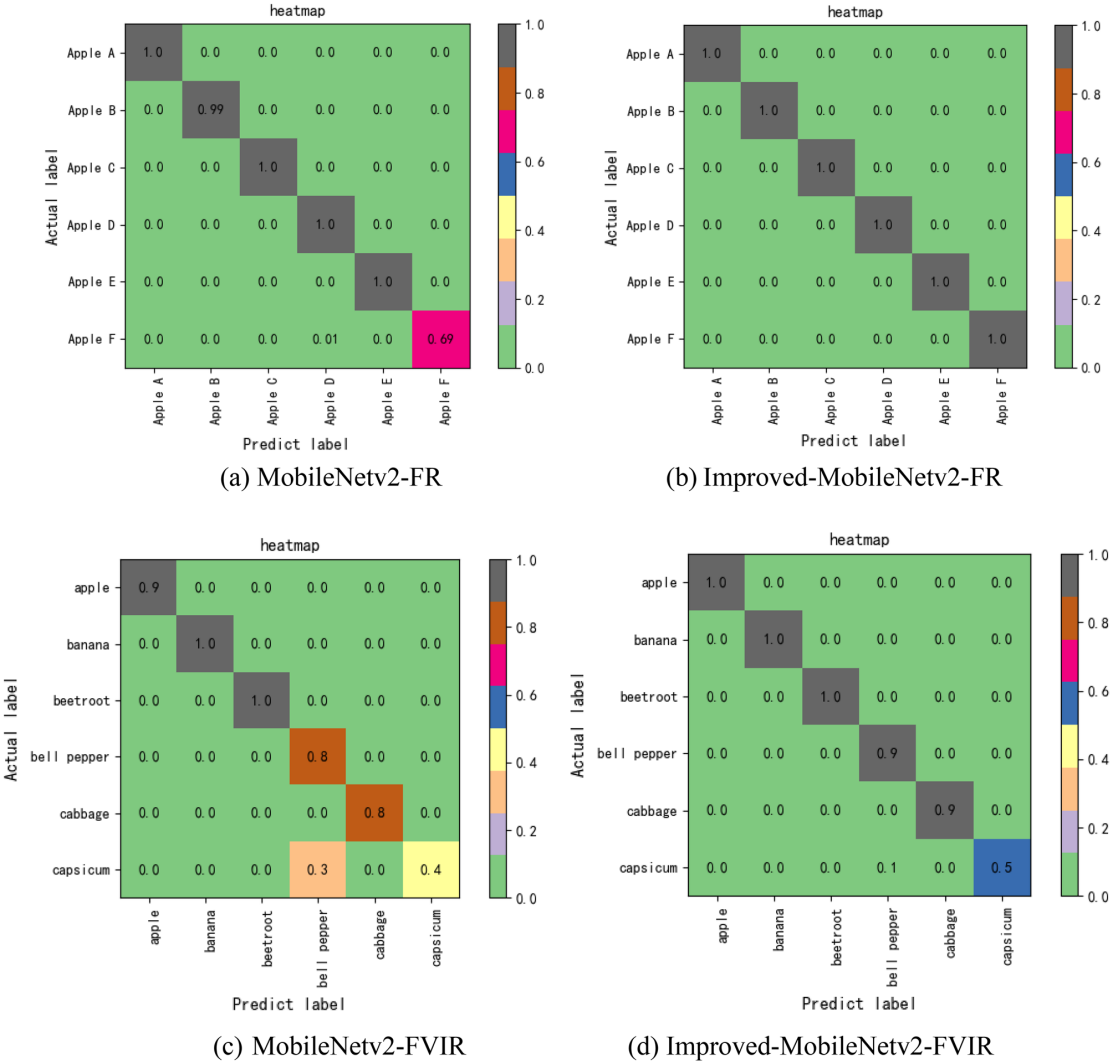
(**a**) Is the confusion matrix tested by MobileNetv2 on the fruit recognition dataset, (**b**) is the confusion matrix tested by MobileNetv2 on the fruits and vegetables image recognition dataset, (**c**) is the confusion matrix tested by Improved-MobileNetv2 confusion matrix tested on the fruit recognition dataset, and (**d**) is the confusion matrix tested on the fruits and vegetables image recognition dataset by Improved-MobileNetv2.

As there are so many different types of fruits and vegetables in the dataset, we chose the first six categories as representatives to produce a confusion matrix to show the effect of different models on the recognition of the dataset, as shown in Fig. 10.

#### Comparison the results of different models with experiments

To evaluate the classification performance of the improved MobileNetv2 model, we selected four other models for comparison: AlexNet, VGG16, ResNet34, and ResNet50. The performance of each model was compared by evaluating their F1 scores, accuracy, and the number of parameters, as shown in Table 4.

**Table 4:** Comparison of the accuracy of different models.

	ResNet34	ResNet50	VGG16	AlexNet	Improved-MobileNetv2
F1	0.92	0.93	0.96	0.97	0.99
Accuracy	97.21%	97.45%	98.60%	98.03%	99.96%
Parameters	21,350,915	23,856,131	70,781,891	62,378,344	3,028,211

In this table, we compared the performance metrics of different models, including F1 score, accuracy, and the number of parameters. Considering five different models, namely ResNet34, ResNet50, VGG16, AlexNet, and the improved MobileNetv2 model. The F1 score considers the balance between precision and recall, and the improved MobileNetv2 model performed the best in this regard, with the highest F1 score (0.99), followed by VGG16 and AlexNet with F1 scores of 0.96 and 0.97, respectively. ResNet34 and ResNet50 had F1 scores of 0.92 and 0.93, respectively.Accuracy reflects the proportion of samples correctly classified by the model. Here, the VGG16 model performed the best with an accuracy of 98.60%, followed by AlexNet (98.03%) and ResNet50 (97.45%), while ResNet34 and the improved MobileNetv2 had accuracies of 97.21% and 99.96%, respectively. The number of parameters in the model indicates its complexity, and generally, fewer parameters are preferred because fewer parameters usually mean a more lightweight model. The improved MobileNetv2 model had the fewest parameters (3,028,211), while VGG16 and AlexNet had more parameters (70,781,891 and 62,378,344, respectively).

**Figure 11:**
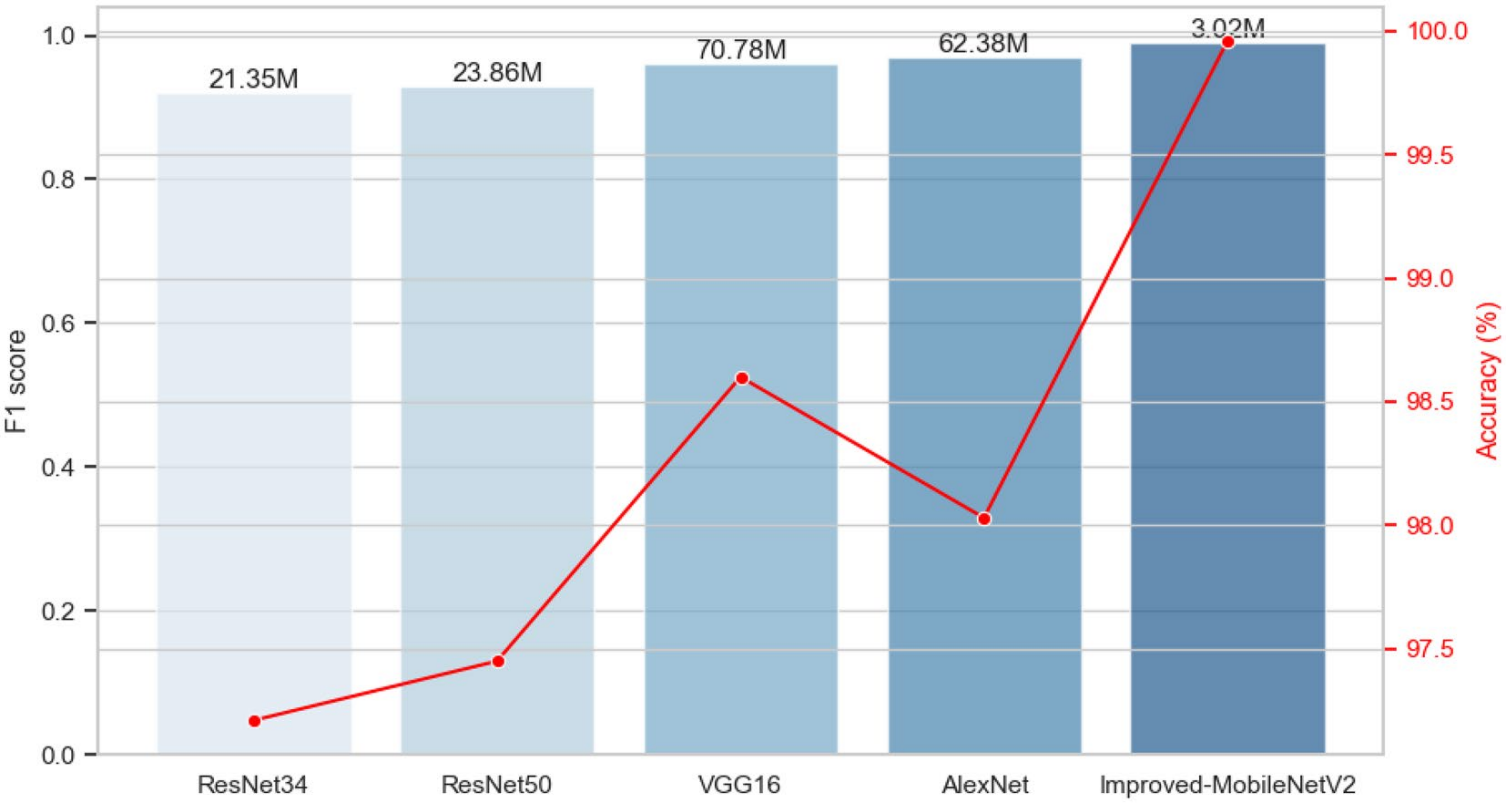
Performance comparison of different models.

Overall, the improved MobileNetv2 model excels in both F1 score and accuracy while having fewer parameters, making it advantageous in terms of performance and model lightweightness. It's worth noting that AlexNet has a relatively shallow network structure, consisting of only 5 convolutional layers and 3 fully connected layers, whereas VGG16 has a very deep network structure, comprising 16 convolutional layers and 3 fully connected layers. The innovation of ResNet (Residual Network) lies in the introduction of "residual blocks," with ResNet34 and ResNet50 containing 34 layers and 50 layers of ResNet networks, respectively. Compared to ResNet34, ResNet50 adds additional convolutional layers and residual blocks. In contrast to these models, the improved MobileNetv2 model not only achieves higher accuracy but also has fewer parameters. Figure 11 illustrates the comparative results of these models.

## Conclusions and future work

Given the diverse nature of agricultural products and the presence of similar subcategories, which can lead to accuracy issues in classification, this paper proposes an improved MobileNetv2 agricultural product recognition model. By innovatively introducing Res-Inception and EMA, this model significantly enhances accuracy in agricultural product classification tasks. The improved MobileNetv2 network demonstrates outstanding performance and can be widely applied in the agricultural product classification field. Future research directions will focus on how to utilize image augmentation techniques for data augmentation while reducing network parameters, all while maintaining accuracy, to further enhance classification accuracy. This will help make the model more efficient and suitable for a broader range of agricultural product classification tasks.

## Data Availability

The datasets generated during and/or analyzed during the current study are available from the corresponding authors upon reasonable request.
